# Gambling adverts on social media reach 2.3 times more men than women: Using the Meta Ad library to assess gambling advertising in Ireland

**DOI:** 10.1556/2006.2025.00484

**Published:** 2026-04-28

**Authors:** Elena Petrovskaya, Leon Y. Xiao, Nicole Khoo, Deirdre Leahy, Amanda Roberts

**Affiliations:** 1 Department of Computer Science and Technology, University of Cambridge, UK; 2 College of Health and Science, University of Lincoln, UK; 3 School of Creative Media, City University of Hong Kong, Hong Kong, China; 4 beClaws.org, UK; 5 School of Arts and Creative Industries, London South Bank University, UK; 6 School of Law, University College Cork, Ireland; 7 School of Computer Science, Munster Technological University, Ireland; 8 School of Sport, Exercise and Health Sciences, University of Loughborough, UK

**Keywords:** gambling, advertising regulation, consumer protection, Ireland, EU digital services act, transparency, social media

## Abstract

**Background and aims:**

Gambling is a public health issue, leading to many harms. Gambling advertising is prevalent and constant, including on social media, which allows for personalised targeting and wide reach. Our aim was to understand the reach and target demographics such advertising.

**Methods:**

The EU Digital Services Act now requires large online platforms to provide repositories for all advertising shown and associated audience demographic data for EU countries. We used Ireland as a case study to assess the scale of targeting specific genders and age groups by gambling companies, as well as the actual reach of the adverts. We checked through the publicly available list of remote betting licensees operating in Ireland (*n* = 88). Adverts were examined as to their intended target demographics and actual impressions by various demographic groups.

**Results:**

Between January and February 2025, we studied 411 adverts across 88 operators. Ninety-one adverts (22%) targeted men only; no adverts (0%) targeted only women. 12,690,245 men were reached across all the 411 adverts, 2.3 times more compared to 5,458,438 women. The age group of 25–34 reached the most unique accounts, with a total of 6,246,408 accounts reached (33.9% of all accounts reached).

**Discussion and Conclusions:**

These findings illustrate male-skewed delivery in gambling advertising in Ireland, and demonstrate the utility of platform ad repositories for addiction research.

## Introduction

Gambling advertising is commonplace. Exposure to such advertising is linked to a positive association with gambling-related attitudes, intentions, and behaviours (Bouguettaya et al., 2020), potentially because it triggers impulses to gamble (Hing, Cherney, Blaszczynski, Gainsbury, & Lubman, 2014). Thirty percent of current gamblers (in the UK, 2019–2020) reported unplanned gambling expenditures after seeing a gambling advert, promotion, or sponsorship (Wardle et al., 2022). Social media facilitates the promotion of gambling in less obvious ways, and with more targeting (Rossi & Nairn, 2022), which leads to the normalisation of gambling (Gainsbury, Delfabbro, King, & Hing, 2016). Such advertising is widespread, meaning exposure to gambling practices and brands is constant (Thomas et al., 2023). Social media advertising is also more time-sensitive due to its ability to be more flexible and focus on upcoming or current events, thus creating artificial urgency in the gambling behaviour itself (Rossi, Nairn, Smith, & Inskip, 2021). Gambling advertising on social media has increased in recent years (Wardle et al., 2024), branching into new and subtle types such as influencer-driven marketing (Bolat et al., 2025). Engagement with such advertising is linked to moderate risk and disordered gambling: gamblers in those categories are more likely to report that they had increased their gambling because of these promotions (Gainsbury, King, et al., 2016). Such advertising also taps into impulsivity in consumers aged under 25 (Pechmann, Levine, Loughlin, & Leslie, 2005).

Ireland is a country with a strong gambling culture: 64.5% of people in Ireland reported being engaged in various forms of gambling in the previous year, and of those, an estimated 40,000 are high-risk gamblers (Kerr, O'Brennan, & Vazquez Mendoza, 2021). This is higher than the global prevalence, which in 2024 was 46.2% (Tran et al., 2024). As per the above, certain types of gambling advertising can influence gambling behaviours, most likely through a ‘dose-response’ effect, where greater advertising increases gambling participation, leading to a greater risk of harm (McGrane et al., 2023). Simultaneously, gambling advertising can have enormous reach. However, a comprehensive evidence base investigating gambling reach across various countries and regulatory jurisdictions is lacking, as is methodology which allows for an objective assessment of paid advertising content promoted by operators which may contribute to this reach (Singer, Wöhr, & Otterbach, 2024).

Young people are exposed to gambling marketing through sports consumption, television, and social media (Kerr et al., 2024). This is particularly prevalent in the case of live sports, as sports companies have built brands and communities directly connected to gambling (Bidav, McEvoy, Kitchin, Kerr, & O'Brennan, 2024). Gambling is normalised as a part of sports fandom for male youths (McGee, 2020). Football, in particular, is particularly subject to ‘gamblification’, with gambling marketing deeply connected to many Premier league football clubs (Lopez-Gonzalez & Griffiths, 2018). Sports-related gambling adverts offer ‘free bets’ (promotional offers to encourage new players to sign up) as well as in-play promotions (betting on a sport while it is occurring), both of which are significant inducements (Newall, Cortis, & Torrance, 2025). The link between sports and gambling, including in sports-related gambling adverts, may translate into advertising content which is particularly appealing to young males. Gambling sponsorships are one of the most prevalent forms of gambling advertising (De Jans, Hudders, & Constandt, 2024), and qualitative research with men aged 20–37 shows that gambling marketing related to sports serves to normalise gambling (Deans, Thomas, Derevensky, & Daube, 2017). This link between sports and gambling makes gambling marketing a dangerous pipeline for this demographic—especially combined with direct targeting of men by operators.

Particularly for young men, there are a range of motivational factors for sports betting engagement, which include making money, minimising boredom, and appearing more knowledgeable about sports (Nyemcsok, Pitt, Kremer, & Thomas, 2022). Indeed, young men, aged 18–30, are a particularly high-risk group for gambling harms (Hing, Russell, et al., 2016). In Ireland, males aged 25–34 years have the highest rate of high-risk gambling (1.3%) compared to other genders and age groups: in the same age category, females have a rate of only 0.2% (Mongan, Millar, & Galvin, 2021). Such a trend is also observed among younger males, including teenagers: when controlling for gambling participation in the past year, the prevalence of high-risk gambling in males aged 15–24 was 1.5%, and at-risk gambling was 18% (Mongan et al., 2021).

It remains unclear, however, whether companies are consciously and consistently targeting younger men with gambling advertising. Prior research studying exposure to gambling advertising in groups of interest has used primarily measures of self-report to assess this exposure. For instance, Di Censo, Delfabbro, and King (2024), conducted an analysis of 19 studies on gambling advertising exposure and young people. Their results indicated that the majority of the studies in their sample used self-reported exposure or recall. These recall-based methods inevitably incorporate human bias, as well as relying on (imperfect) human memory, making it difficult to draw conclusions around how prevalent such targeting truly is.

A more objective assessment of the targeting of gambling social media advertising is possible using newly available ad repositories, which contain accurate and objective data. Article 39 of the recently adopted EU Digital Services Act (DSA) [2022] OJ L277/1 now requires large online platforms to provide repositories for all advertising shown and associated audience demographic data (*e.g.*, which age group was targeted and how many male users were reached) for EU and EEA countries. This includes the Meta Ad Library (https://www.facebook.com/ads/library/), which publishes advertising-related data for Meta-owned platforms, including Facebook, Instagram, and Messenger. This resource can be used to objectively sample and study commercial advertising on social media (Xiao, 2025; Xiao, Petrovskaya, Khoo, Denoo, & Roberts, 2025). As such, as well as assessment of the targeting of specific demographics by gambling operators in paid advertising, it provides a useful way to study the prevalence, content and reach of such advertising.

### Gambling in Ireland

Ireland is a country that provides an ideal case study for the use of the Meta Ad Library to study advertising. First, Ireland is an EU country, so Meta is legally obliged by the DSA to provide national data. Second, advertisements published in Ireland are mostly in English and so can be more easily studied by international researchers. As discussed above, gambling is common in Ireland, and many individuals engage in high-risk gambling, which lends value to studying the specifics of gambling advertising in Ireland (Mongan et al., 2021; Kerr et al., 2021).

At the time of the conceptualisation and execution of the study, Irish gambling regulation was covered by several laws, which focused primarily on different aspects of gambling, such as betting and lotteries (e.g. 2013 National Lottery Act), with no one coherent regulation or regulator designed to protect consumers. Advertising, including social media advertising, was subject only to rules through self-regulation by the Advertising Standards Authority, which meant ads had to encourage responsible gambling and not promote harmful gambling.

However, the study also took place at a core transitional moment for Irish gambling legislation. The Gambling Regulation Act 2024 was enacted in October 2024, with its provisions being commenced in phases from 5 March 2025, when the Gambling Regulatory Authority of Ireland (GRAI) was formally established. Among other measures, the Act provides for a Social Impact Fund to finance initiatives aimed at raising awareness of, and reducing, harmful gambling. It also grants the GRAI extensive powers to introduce further regulations regarding advertisements (Section 144), and to oversee and enforce compliance. Section 149 introduces a watershed, prohibiting gambling advertising on broadcasting, audio-visual on-demand media services, and on-demand sound services between 5:30 AM and 9:00 PM. Gambling advertising via social media and video-sharing platforms will be restricted to account holders who follow a licensed gambling operator (Section 146). In addition, Section 147 prohibits advertising distributed by email, unless the recipient has consented to such communications.

While we used Ireland as a case study for the current research, due to the pre-regulation context in which the adverts were published, the research and methodology are applicable internationally. The gambling industry is growing worldwide, and gambling is currently legal in more than 80% of all countries (Wardle et al., 2024). Gambling advertising on social media is currently allowed in many other regions, including the United Kingdom, Australia and sub-Saharan Africa (Kallman & Wyllie, 2024; Badu, 2024). Ireland is a useful case study for a country where gambling regulation is regulated in a comparatively liberal way, and a high number of individuals gamble, with an estimated 3.3% of the population falling into the high-risk gambling category (Ceallaigh, Timmons, Robertson, & Lunn, 2023): this description is applicable to many other countries, and the findings on paid social media advertising, which can be seen by users regardless of whether they consented to this advertising, will therefore likely be of interest to regulators internationally (particularly in Europe, where the same regulations apply regarding advertising transparency). Understanding the landscape of reach, targeting, and content in social media advertising in Ireland, therefore, provides a useful picture for similar situations in other countries, and our proposed methodology is applicable to studying similar research questions worldwide.

In the current research, we sought to answer two research questions (which were preregistered at https://doi.org/10.17605/OSF.IO/Q4CDU) using the Meta Ad Library as an objective and independent source of gambling advertising-related data.

Research Question 1: What are the demographics being targeted by gambling and betting companies in Ireland?

Research Question 2: What are the actual reach figures for the targeted demographics?

## Methodology

The Meta Ad Library was used as the source of the adverts. Between January and February 2025, one researcher systematically checked through the publicly available official list of remote betting licensees operating in Ireland (Supplementary material), which included adverts active between March 2024 and the time of search. No other operators were included. There were no other inclusion or exclusion criteria. The Meta Ad Library includes adverts posted on all Meta-owned platforms, including Facebook, Instagram and Messenger: these were not differentiated between in our analysis.

The list contained 88 companies. The researcher checked for trading name and licensee name, and, in some cases, cross-referenced with the company's Facebook page to make sure the right page had been located, particularly the case in more ambiguous brand names such as Matchbook and Spreadex.

**Fig. 1.**
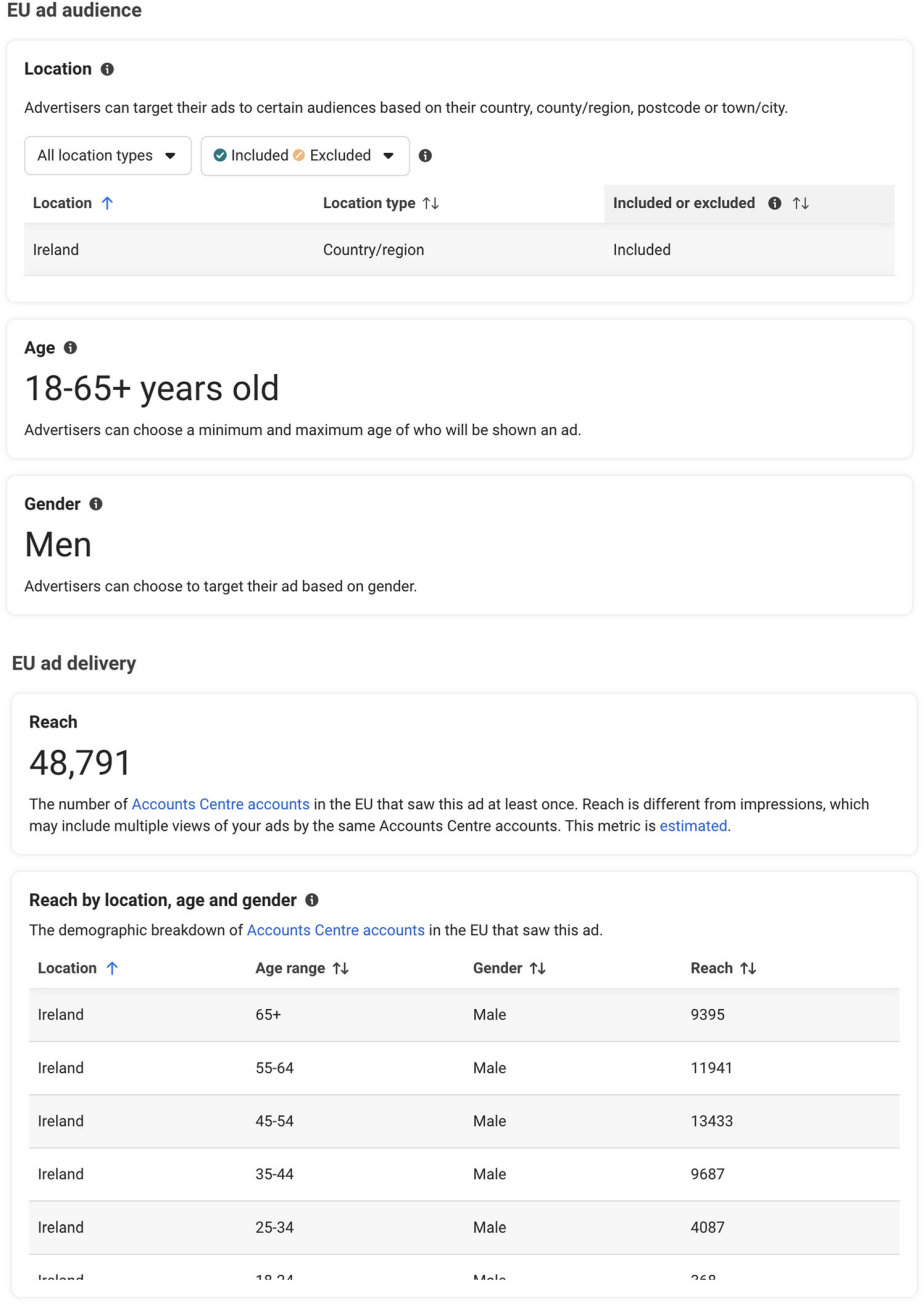
An example of how the advertising information for each advert is presented on the advert's page in the Meta Ad Library

Once the page had been found, the most recent 20 ads (or fewer, if only fewer were available) published by each licensee were studied for their intended target demographics, and actual impressions by various demographic groups (such as gender and age). This was done by checking the relevant categories, which are provided on the advert's page according to the Meta Ad Library guidelines, as illustrated in Fig. 1. Both active and inactive adverts were included: active adverts were running on social media at the time of data collection, whereas inactive adverts had concluded and were no longer being shown to users. Twenty posts only per operator were included in the analysis to afford balance between investment of resources and time, while still being a sufficient sample to paint a picture of the per-operator targeting trends.

All ads and associated information were screenshotted and deposited on the OSF (https://osf.io/zjkcq/overview?view_only=80ffbc71db0d405585bbd464cc2e2bb0).

We did three rounds of inter-rater reliability calculation with a second rater to check both for the presence of advertising pages (whether an operator had a page at all, to ensure no adverts had been missed in the analysis) and the reach numbers in the adverts. However, in each round, the inter-rater reliability was below 0.8, which was not sufficiently satisfactory (for advertising numbers, such as reach, Krippendoff's alpha was *a* = 0.59, 0.79 and 0.69, and for the presence of operator advertising pages, Cohen's Kappa was 0.41, 0.31 and 0.68), which we believe to be due to the difficulty of transferring numbers manually from the platform. The first author subsequently checked through the entire dataset, using screenshots taken at the original time of data collection, and corrected any initial errors in data collection.

### Ethics

No human participants or personal data were collected in this research, and the University of Lincoln Human Research Ethics Committee confirmed ethical approval was not required. All data were obtained from a public repository; no interaction with platform users occurred.

## Results

### Research Question 1: What are the demographics being targeted by gambling and betting companies in Ireland?

Of 88 licensed operators, 64 (73%) had a Meta Ad Library page, and 24 (27%) had one or more adverts on their pages. Operators which had published 1+ advertisements were included in the analysis. The total number of adverts studied was 411.

Gender-wise, of those 411, 91 adverts (22%) targeted men only. A further 16 did not set any target. The rest, 304 adverts (74%), set their targeting to ‘All.’ No adverts (0%) targeted only women.

Age-wise, the majority of adverts (*n =* 372, 91%) listed the upper age as 65 or as 65+. One exception was LeoVegas Gaming PLC (LeoVegas), which had eight adverts set to target the age group ‘25–45,’ and another was LiveScore Bet, which had 13 adverts targeting the ‘35–54’ age group. One hundred and eighty adverts (44%) set the lowest age limit to be 18, meaning they encapsulated the vulnerable group of users aged ‘18–24.’ Of those 180, 55 ads (31%) additionally targeted men only. Three hundred and ninety-eight adverts included some of the age group between 18–34 in their targeting. The full breakdown of age targeting is shown in Table 1.

**Table 1.** Breakdown of targeting of gambling advertising by age (*N* = 411)

Target age	Frequency
18–55	1 (0.2%)
18–64	1 (0.2%)
18–65+	178 (43.3%)
20–65	3 (0.7%)
21–65	16 (4.0%)
25–45	8 (19.5%)
25–65+	165 (40.1%)
30–65+	10 (2.4%)
35–54	13 (3.2%)
Did not target	16 (3.9%)
**TOTAL**	411

### Research Question 2: What are the actual impressions reached for the targeted demographics?

Notably, ‘reach’ refers to the number of unique accounts that saw an advert at least once (Meta, 2025). This does not account for multiple accounts created by the same person viewing the ad (which would be counted multiple times), and it does not include multiple views by the same account (which would only be counted once).

In total, the adverts in the sample reached 18,389,653 accounts (note: between adverts, there is no way to know whether the same individual is being reached several times on the same platform, nor how many times this occurred).

Although only 22% of adverts studied targeted men directly (*n* = 91), 12,690,245 men were reached across all of the 411 adverts, compared to only 5,458,438 women: *i.e.*, 2.3 times more men than women were reached by the same ads. The majority of this reach came from adverts that did not target men directly: 10,964,302 men were reached across adverts targeting ‘All’ or not specifying a target. A further 260,822 users of unknown gender (1.42%) were reached.

The age group that was reached the most by the adverts across all genders was ages 25–34, in which 6,246,408 accounts were reached (33.9%). The second most-reached group was ages 35–44, reaching 4,701,885 accounts (25.5%). Further reach details are shown in Table 2.

**Table 2.** Breakdown of the reach of gambling advertising by age and gender

	Male	Female	Unknown	Total
18–24	1,394,683 (7.4%)	443,736 (2.4%)	68,667 (0.4%)	1,907,086 (10.3%)
25–34	4,520,017 (24.5%)	1,661,662 (9%)	64,729 (0.4%)	6,246,408 (33.9%)
35–44	3,160,860 (17.2%)	1,487,032 (8.1%)	53,993 (0.3%)	4,701,885 (25.5%)
45–54	2,019,578 (11.1%)	1,009,827 (5.5%)	44,724 (0.2%)	3,074,129 (16.7%)
55–64	1,023,088 (5.6%)	525,816 (2.9%)	17,441 (0.1%)	1,566,345 (8.5%)
65+	572,019 (3.1%)	330,365 (1.8%)	11,268 (0.06%)	913,652 (5%)
*Total*	**12,690,245** (68.7%)	**5,458,438** (29.6%)	**260,822** (1.42%)	**18,389,653**

The single ad (https://www.facebook.com/ads/library/?id=831898489123277) with the most reach—published by BetFair—reached 1,320,179 unique Meta accounts in Ireland, equalling 26% of the total Irish population, which is 5.15 million (An Phríomh-Oifig Staidrimh, 2023). The ad in question featured a description of Betfair's safer gambling tools - alongside footage of gambling, such as slots. Out of the 5 most viewed ads in the sample, Betfair accounted for three, and BetVictor for the other two. Taken together, these 5 most viewed ads alone reached 3,688,413 accounts. This demonstrates the reach of gambling advertising even across a small number of gambling companies and individual ads.

## Discussion

We analysed 411 adverts from a sample of 88 Irish operators with remote betting licences using the Meta Ad Library, which allows objective access to such adverts and their associated demographic data. Out of the list of 88 operators, only 24 had a page with any adverts on the repository. The 411 ads we studied were only a small portion of all ads published by these gambling licensees. However, the reach across even these few published adverts was very high, a total of 18,389,653 unique users, and the single advert with the highest reach was viewed by 3,688,413 accounts at least once.

### Males and young people

Explicit gender targeting was infrequent (22% men-only; 0% women-only), yet delivery combined to produce a 2.3× male reach. This pattern suggests that ad delivery algorithms yield strong male exposure even without heavy use of explicit male-only targeting. Fifty-five adverts (13%) set the lowest age limit of targeting to be 18 and also targeted men directly. The age range of 18–35 was included in the age targeting by 398 ads. We also found that the age group of 25–34 reached the most unique accounts, with a total of 6,246,408 accounts reached: this made up 33.9% of total accounts reached. The second most-reached group was ages 35–44, reaching 4,701,885 accounts (25.5%). This means the adverts targeting the group aged 25–44 reached 59.4% of all accounts reached: a clear majority.

The above findings are particularly interesting in the context of Irish gambling: males under 35 are more likely to bet, either in a bookmaker's shop or online, and males in Ireland have a significantly higher prevalence of problem gambling than females (1.4% vs. 0.2%) (Kerr et al., 2024). Males aged 25–34 years have the highest rate of problem gambling (1.3%), and males aged 35–49 were the most likely to report gambling in the past year (60%) (Mongan et al., 2021). This suggests a relationship between operator targeting of this group and the prevalence and harms from gambling: either operators are aware of this vulnerability, or this group might experience harm in some way linked to intentional increased advertising presence. Online gambling in Ireland has also been seen to increase between the ages of 17–20 (Duggan & Mohan, 2023), particularly in males—this group was not with the highest reach in our sample, but it is possible that in the context of longer-term gambling behaviours, they would be more at risk of gambling harms when they reach the group of 25–34.

### Recommendations and future work

The empirical data for this study were collected in January and February 2025. Noting that since March 2025, according to the Gambling Regulation Act, gambling advertisements on social media are prohibited unless the user actively consents to receiving them, the current reach of adverts should theoretically now be lower as provided in the Gambling Regulation Act. That said, it may be possible that operators continue to advertise illegally, just as video game companies are known to do in Belgium in contravention of regulation (Xiao et al., 2025). This suggests that the enforcement of the advertising provisions by the GRAI will be key to assessing the impact of the Gambling Regulation Act. Future work should investigate this in the Irish context once the Act has had time to come into force.

As mentioned above, the new Gambling Regulation Act 2024 came into force in March 2025, after our data collection was complete. Because of this, we were unable to comment on the impact of the new legislation on advertising practices, nor was it within the scope of our study. We could not use the Meta Ad Library to determine whether any users reached were existing subscribers of the relevant gambling account. Future work can therefore assess the effectiveness of the Act and the compliance of gambling companies and social media organisations with their respective obligations under the Gambling Regulation Act and the DSA. In future work, involvement of the Advertising Standards Authority for Ireland as a regulatory partner would also add a further dimension to the assessment of the impacts and effectiveness of the Irish frameworks around marketing communications for gambling in Ireland (Gambling Regulatory Authority of Ireland, 2025).

### Limitations

Despite the general reliability of the data on Meta Ad Library given the legal requirements around storing advertising data on this platform imposed by the DSA, there are certain issues with using this platform. One example is the lack of usability of the search function for specific word strings, which does not produce exclusively the page of interest but sometimes unrelated adverts. The easiest way to navigate this issue is to include the page ID in the dataset so that the correct page is being verified by inter-raters.

The Meta Ad Library also has a history of removing adverts when they have blatantly violated advertising standards by including prohibited content: this happened to 4 adverts in our sample for which we could not access the content of the advert—although we could still see the targeting and reach. Though it did not affect the data of this study, given that we do not claim to have completed a comprehensive analysis of every gambling advert published on the platform, it may be a noteworthy issue to consider when using this platform as a data source for further studies.

There have also been discrepancies between the total reach calculated with the demographic breakdowns and the total reach reported by the Library, and the reasons for this cannot be verified due to the lack of reporting of how the platform arrived at the latter number. This is indicative of the larger limitation of using the Meta Ad Library for research, which is that the data on the platform cannot be independently verified by researchers.

Finally, we acknowledge that our sample constitutes a small proportion of all gambling adverts published on social media: we cannot establish exactly what the proportion is within the scope of this study and thus are not able to generalise our numerical findings across all gambling advertising. Similarly, the data was collected manually, and it is not impossible (although highly unlikely, given our stringent data verification process) that minimal inaccuracies are present.

## Conclusion

An analysis of 411 adverts sampled between January and February 2025 across 88 operators with remote betting licences that operate in Ireland showed 91 adverts (22%) targeted men only, with no adverts targeting only women. Across all 411 adverts, 12,690,245 men were reached, compared to 5,458,438 women. In total, adverts targeting some part of the age group 25–44 reached 59.4% of all accounts reached. This analysis of adverts points to an advertising landscape actively aiming to recruit male gamblers and demographics known to have high prevalence of gambling and high-risk gambling.

We also hope that stakeholders can appreciate the value of and make further use of the Meta Ad Library as an objective data source that is legally required to detail demographic targeting and actual reach of unique accounts of advertising in the EU and EEA countries. This means advertising both across gambling and other harmful industries can be scrutinised by independent researchers to advise policymaking (including implementation and compliance), and we encourage others to address many other important research questions using this tool to demonstrate to policymakers that laws similar to the DSA should be more widely adopted to provide transparency and accountability.

## Supplementary material

## Data Availability

All advertising materials analysed, the raw data, and the analysis script and output are publicly available in the Open Science Framework at http://doi.org/10.17605/OSF.IO/ZJKCQ.
