# Supplementary material for: Gambling adverts on social media reach 2.3 times more men than women: Using the Meta Ad library to assess gambling advertising in Ireland
Source: J Behav Addict. 2026 Apr 28;15(2):824–32. doi: 10.1556/2006.2025.00484 (PMC13371774; doi:10.1556/2006.2025.00484)
Supplement: Supplementary file 1 [file jba-15-824-s001.pdf]

Petrovsakya, E. et al.: Gambling adverts on social media reach 2.3 times more men than women: using the Meta Ad Library to assess gambling advertising in Ireland. https://doi.org/10.1556/2006.2025.00484

Supplementary material: The full list of licensed remote bookmarking operations in Ireland

List of Current Licensed Remote Bookmaking Operations
(in accordance with relevant legislation) @ 01 March 2024
Please Note: Other Operators may have obtained their licences since this date but will not be reflected here

Licence Ref	Description	Licensee Name	Trading Name	Nominee Name	Address 1	Address 2	Address 3	Address 4	Address 5
1020537	Remote Bookmaker's Licence	TRACK SPORTS LTD	NA	MR PAUL REILLY, MR JAMES ANTHONY REILLY	27 DROGHEDA STREET	BALBRIGGAN	CO DUBLIN	CO. DUBLIN	NA
1010121	Remote Betting Intermediary's Licence	TRIPLEBET LIMITED	MATCHBOOK	. GARRON JERI WHITESMAN, . HELEN ST. CLAIR ACKRILL	INCHALLA	LE VAL	ALDERNEY	N/A	GY9 3UL
1010114	Remote Bookmaker's Licence	TSG INTERACTIVE GAMING EUROPE LIMITED	POKERSTARSSP ORTS	DANIEL TAYLOR, MR KEVIN FRANCIS HARRINGTON	SPINOLA PARK LEVEL 2	TRIQ MIKIEL ANG BORG	ST JULIANS SPK 1000	N/A	MALTA
1011286	Remote Bookmaker's Licence	BML GROUP LIMITED	BETSAFE	. PETER DOUGLAS THURSTON, . OLGA FINKEL	EXPERIENCE CENTRE TA' XBIEX SEAFRONT	TA' XBIEX	TXBX1027	N/A	MALTA
1010150	Remote Bookmaker's Licence	CELTON MANX LIMITED	NA	. CECIL SOO JIN SNG, . WILLIAM DAVID MUMMERY	CELTON HOUSE	DOUGLAS	ISLE OF MAN	N/A	ISLE OF MAN IM2 2QZ
1010233	Remote Bookmaker's Licence	BV GAMING LIMITED	BETVICTOR / HEARTBINGO / TALKSPORT BET	. ERIC POULTON, . ROBERT SMITH, . ANDREAS MEINRAD	SUITE 23 PORTLAND HOUSE	GLACIS ROAD	GIBRALTAR GX11 1AA	N/A	GIBRALTAR

Licence Ref	Description	Licensee Name	Trading Name	Nominee Name	Address 1	Address 2	Address 3	Address 4	Address 5
1010277	Remote Bookmaker's Licence	RACEBETS INTERNATIONAL GAMING LIMITED	NA	. PETER DOUGLAS THURSTON, . OLGA FINKEL	DRAGONARA BUSINESS CENTRE	DRAGONARA ROAD	ST JULIAN'S STJ 3141	N/A	MALTA
1010329	Remote Bookmaker's Licence	NETBET ENTERPRISES LIMITED	NETBET	. DARREN MERCIECA, . ALINA GABRIELA ARNAUTU	EWROPA BUSINESS CENTRE	LEVEL3 SUITE 704	BIRKIRKARA BKR9034	N/A	MALTA
1016820	Remote Bookmaker's Licence	LAN DECRAN LIMITED	MACAU SPORTING CLUB	MR FINBARR CORKERY	16 PATRICKS QUAY	CORK	CO CORK	CO. CORK	NA
1010701	Remote Bookmaker's Licence	INTERWETTEN GAMING LTD	NA	. MICHAEL BLUEMEL, . WOLFGANG FABIAN	2ND FLOOR, LIFESTAR BUILDING	TESTAFERRATA STREET	TA'XBIEX XBX 1403	N/A	MALTA
1011284	Remote Bookmaker's Licence	EU LOTTO LTD	LOTTOLAND	. MATTHEW ROBINSON, . NIGEL BIRRELL	SUITE A	OCEAN VILLAGE PROMENADE	OCEAN VILLAGE	N/A	GIBRALTAR GX111A
1011600	Remote Bookmaker's Licence	MR GREEN LIMITED	MR GREEN	. STEPHEN BELL, . ALEXIS ZAMBOGLOU	14 TAGLIAFERRO BUSINESS CENTRE	HIGH STREET	MALTA SLM 1549	N/A	MALTA
1012453	Remote Bookmaker's Licence	LEOVEGAS GAMING P.L.C.	LEOVEGAS, BETUK.COM, 21.CO.UK	. CARL MATTIAS WEDAR, . STEFAN NELSON	LEVEL 7 THE PLAZA CENTRE	BISAZZA STREET	SLIEMA SLM 1640	N/A	MALTA
1019738	Remote Bookmaker's Licence	SKS365 MALTA LIMITED	SKS365 MALTA LIMITED	. TROY MICHAEL COX, . ANDREW NAUDI	135 CENTRAL BUSINESS CENTRE	LEVEL 2, SPINOLA BAY	ST JULIANS STJ 3095	N/A	MALTA

Licence Ref	Description	Licensee Name	Trading Name	Nominee Name	Address 1	Address 2	Address 3	Address 4	Address 5
1018032	Remote Betting Intermediary's Licence	EXCHANGE PLATFORM SOLUTIONS LIMITED	BETDAQ	. HENRY SMITH	INCHALLA	LA VAL	ALDERNEY GUERNSEY	N/A	UK
1018155	Remote Bookmaker's Licence	PROZONE LIMITED	BETHARD	. FRANK HEINANEN, . ERIK JOHAN SEBASTIAN SKARP	MEZZANINE OFFICE	THE GEORGE, TRIQ BALL	ST JULIANS STJ 3123	N/A	MALTA
1019644	Remote Bookmaker's Licence	QUINNBET (GIBRALTAR) LTD	QUINNBET	MR STEPHEN KELLY, MISS BRENDA QUINN	MADISON BUILDING	MIDTOWN	QUEENSWAY, GX111AA	N/A	GIBRALTAR
1020370	Remote Bookmaker's Licence	CONDOR MALTA LTD	CONDORGAMIN G	. HENRIK JOZSEF PISKI, . MARIJANA ZECEVIC	13 PAOLO COURT GROUND FLOOR	GIUSEPPE CALI STREET	TA'XBIEX XBX 1423	N/A	MALTA
1020581	Remote Bookmaker's Licence	NAALE LTD	NAALE LTD	. ROGER STRICKLAND, . MYKHAILO LIUBYMOV	LEVEL 3 (SUITE 3068)	TOWER BUSINESS CENTRE TOWER STREET	SWATAR BIRKIRKARA, BKR 4013	N/A	MALTA
1011639	Remote Bookmaker's Licence	PAUL FAY BOOKMAKERS LIMITED	PAUL FAY BOOKMAKERS LIMITED	MS SINEAD FAY, MR PAUL FRANCIS FAY	MAIN STREET	GRANARD	NA	CO. LONGFORD	NA
1010112	Remote Bookmaker's Licence	L C INTERNATIONAL LIMITED	NA	. WILLIAM ROBERT STEWART DUTHIE, NEIL ANDREW COTTER	SUITE 6 ATLANTIC SUITES	EUROPORT AVENUE	GIBRALTAR GX11 1AA	N/A	GIBRALTAR
1010108	Remote Betting Intermediary's Licence	BETFAIR INTERNATIONAL PLC	BETFAIR	. CORMAC O'BRIEN, JEFF POWER	SPINOLA PARK - LEVEL 2	TRIQ MIKIEL ANG BORG	ST JULIAN'S SPK 1000	N/A	MALTA

Licence Ref	Description	Licensee Name	Trading Name	Nominee Name	Address 1	Address 2	Address 3	Address 4	Address 5
1010107	Remote Bookmaker's Licence	PPB COUNTERPARTY SERVICES LTD	PADDY POWER	. CORMAC O'BRIEN, JEFF POWER	SPINOLA PARK - LEVEL 2	TRIQ MIKIEL ANG BORG	ST JULIAN'S SPK 1000	N/A	MALTA
1010111	Remote Bookmaker's Licence	BONNE TERRE LIMITED	SKY BET	. PAUL CAULFIELD, . STEPHEN MICHAEL BIRCH	SKY BET	12 VICTORIA STREET	ALDERNEY GY9 3UF	N/A	CHANNEL ISLANDS
1011982	Remote Bookmaker's Licence	BAR-ONE RACING LTD	NA	MR RICHARD GARLAND, MR BRIAN O'HARE	HAGAN HOUSE	RAMPARTS	DUNDALK	CO. LOUTH	NA
1010144	Remote Bookmaker's Licence	BOYLESPORTS (GIBRALTAR) LTD.	BOYLESPORTS	ORLA BOYLE, MR CONOR GRAY	UNIT 7/8	PORTLAND HOUSE	GLACIS ROAD	N/A	GIBRALTAR
1010159	Remote Bookmaker's Licence	BET-AT-HOME.COM INTERNET LTD	BET-AT-HOME.COM	. MARCO FALCHETTO, . ROLAND AIGNER	CORNERSTONE BUSINESS CENTRE	LEVEL 2 SUITE 1 PJAAZA S16 TA SETTEMBRU MOSTA	MST 1180	N/A	MALTA
1020503	Remote Bookmaker's Licence	VIP SPORTS BET LIMITED	VIP SPORTS BET LIMITED	MR JARED CARTHY, MR NOEL CARTHY	3A BALLINALEA	ASHFORD	NA	CO. WICKLOW	NA
1012449	Remote Bookmaker's Licence	ANNEXIO (JERSEY) LIMITED	LOTTOGO.COM	. MR JOHN SPELLMAN, MR PAUL TELFORD	DE CATERET HOUSE	7 CASTLE STREET	ST. HELIER	N/A	JERSEY JE2 3BT
1014834	Remote Bookmaker's Licence	ASPIRE GLOBAL INTERNATIONAL LTD	AGI	. TSACHI ISAAC MAIMON, . OLGA FINKEL	135 HIGH STREET	SLIEMA	SLM 1549	N/A	MALTA

Licence Ref	Description	Licensee Name	Trading Name	Nominee Name	Address 1	Address 2	Address 3	Address 4	Address 5
1015541	Remote Bookmaker's Licence	HILLSIDE (SPORTS) ENC	BET365	. SIMON CARL BETTELEY, . KAREN SILK	1/2373 LEVEL G QUANTUM HOUSE	ABATE RIGORD STREET	TA' XBIEX	N/A	MALTA
1015523	Remote Bookmaker's Licence	CASUMO SERVICES LIMITED	CASUMO	. IDEN AZZOPARDI, . MATTHEW BORG MANCHE	UNICORN CENTRE	TRIQ II-UQIJA	SWIEQI SWQ 2335	N/A	MALTA
1015903	Remote Bookmaker's Licence	KWIFF LIMITED	KWIFF	. CHARLES NICHOLAS LEE, . MICHAEL GOODE	KWIFF PENTHOUSE	DOMESTICA BUILDING,TRIQ IL-WIED	TA L-MISDA, MSD9023, MALTA	N/A	MALTA
1015927	Remote Bookmaker's Licence	FITZDARES LIMITED	FITZDARES	. BALTHAZAR FABRICIUS, . WILLIAM WOODHAMS	182-184 CAMPDEN HILL ROAD	LONDON	W8 7AS	N/A	UK
1016541	Remote Bookmaker's Licence	FAIRLOAD LIMITED	LV BET	. ATHINA ZISI, . AUGUSTO QUINTANO	115B SUITE 3	OLD MINT STREET	VALLETTA VLT 1515	N/A	MALTA
1016824	Remote Bookmaker's Licence	WILLIAM HILL MALTA PLC	NA	. STEPHEN BELL, . ALEXIS ZAMBOGLOU	14 TAGLIAFERRO BUSINESS CENTRE	HIGH STREET	MALTA SLIM 1549	N/A	MALTA
1018829	Remote Bookmaker's Licence	WE THE BOOKIE LIMITED	WE THE BOOKIE	MR MALCOLM WILKINSON	DEMESNE HOUSE	5-6 IVY TERRACE	TRALEE	CO. KERRY	NA
1017720	Remote Bookmaker's Licence	LIVESCORE BETTING AND GAMING (GIBRALTAR) LIMITED	LIVESCORE BET	. KAMRAN QAYYUM CHAUDHARY, . RICHARD IAN LEASK	7.01 WORLD TRADE CENTER	6 BAYSIDE ROAD	GIBRALTAR GX11 1AA	N/A	GIBRALTAR

Licence Ref	Description	Licensee Name	Trading Name	Nominee Name	Address 1	Address 2	Address 3	Address 4	Address 5
1012945	Remote Bookmaker's Licence	ELECTRAWORKS LIMITED	BWIN, SPORTINGBET AND GAMEBOOKERS	. WILLIAM ROBERT STEWART DUTHIE, NEIL ANDREW COTTER	SUITE 6 ATLANTIC SUITES	EUROPORT AVENUE	GIBRALTAR GX11 1AA	N/A	GIBRALTAR
1013174	Remote Bookmaker's Licence	PLATINUM GAMING LIMITED	UNIBET	. TIM COOK, . GRAHAM NIGEL RAY	4TH FLOOR	WORLD TRADE CENTRE	GIBRALTAR GX11 1AA	N/A	GIBRALTAR
1014479	Remote Bookmaker's Licence	EVOKE GAMING LIMITED	EVOKE GAMING LIMITED	. STEPHEN BELL, . ALEXIS ZAMBOGLOU	14 TAGLIAFERRO BUSINESS CENTRE	HIGH STREET	MALTA SLM1549	N/A	MALTA
1014845	Remote Bookmaker's Licence	HOLLYWOODBETS INTERNATIONAL LIMITED	HOLLYWOODBE TS	. DEREK ANTHONY BALFOUR BROWNE, . BASIL MARTIN THOMAS	PO BOX 277	CLINCH'S HOUSE	LORD STREET DOUGLAS	N/A	ISLE OF MAN
1015737	Remote Bookmaker's Licence	PROGRESSPLAY LTD	NA	. NICHOLAS GATT	SOHO OFFICE	3A PUNCHBOWL CENTER ELIA ZAMMIT ST	JULIAS ST J3154	N/A	MALTA
1016176	Remote Bookmaker's Licence	SPREADEX LIMITED	SPREADEX LIMITED	. DAVID MACKENZIE	CHURCHILL HOUSE, 26-30 UPPER MARLBOROUGH ROAD	ST ALBANS	HERTFORDSHI RE, AL1 3UU	N/A	UK
1017678	Remote Betting Intermediary's Licence	BUDDYBET MALTA LIMITED	BUDDYBET	. SCOTT DIDIER, . MANAL IQBAL	ELITE BUSINESS CENTRE	TREJQA TA BOX BOX	MSIDA MSD 1840	N/A	MALTA
1018033	Remote Bookmaker's Licence	EXCHANGE PLATFORM SOLUTIONS LIMITED	BETDAQ	. HENRY SMITH	INCHALLA	LA VAL	ALDERNEY GUERNSEY	N/A	UK

Licence Ref	Description	Licensee Name	Trading Name	Nominee Name	Address 1	Address 2	Address 3	Address 4	Address 5
1010156	Remote Bookmaker's Licence	BETWAY LIMITED	NA	. ANTHONY JAMES WERKMAN, . RICHARD HOWARD AKITT	9 EMPIRE STADIUM STREET	GZIRA GZR 1300	MALTA	N/A	MALTA
1010120	Remote Bookmaker's Licence	888 (IRELAND) LIMITED	888	. STEPHEN BELL, . ALEXIS ZAMBOGLOU	14 TAGLIAFERRO BUSINESS CENTRE	HIGH STREET	SLM 1549	N/A	MALTA
1010300	Remote Betting Intermediary's Licence	SMARKETS (MALTA) LIMITED	SMARKETS	. BENJAMIN MUSCAT, . JASON TROST	LEVEL 7 THE HEDGE	IR-RAMPA TA'SAN GILJAN STREET	ST JULIANS STJ 1062	N/A	MALTA
1010805	Remote Bookmaker's Licence	STAR RACING LTD	STAR SPORTS, NE-BET.COM, AKBETS.BET, MCBOOKIE	MR BENJAMIN KEITH, RUSSELL CANDLER	255 OLD SHOREHAM ROAD	HOVE	BN3 7ED	N/A	ENGLAND
1010917	Remote Bookmaker's Licence	BLUE STAR PLANET LIMITED	10BET	. VALENTIN DIKOV, . ROY MECKENZIE	DRAGONARA BUSINESS CENTRE 5TH FLOOR	DRAGONARA RD, ST JULIANS	STJ2141	N/A	MALTA
1011431	Remote Bookmaker's Licence	32 RED LIMITED	32 RED	. TIM COOK, . GRAHAM NIGEL RAY	4TH FLOOR	WORLD TRADE CENTRE	GX11 1AA	N/A	GIBRALTAR

Licence Ref	Description	Licensee Name	Trading Name	Nominee Name	Address 1	Address 2	Address 3	Address 4	Address 5
1019772	Remote Bookmaker's Licence	ABBEYCOOLE LIMITED	BAMBURY BOOKMAKERS	MR COLM FINLAY	LOWER MAIN STREET	KILBEGGAN	NA	CO. WESTMEATH	NA
1016422	Remote Betting Intermediary's Licence	BOYLESPORTS COUNTERPARTY LIMITED	BOYLESPORTS	ORLA BOYLE, MISS JENNA BOYLE	SUITE 23	PORTLAND HOUSE	GLACIS ROAD GX11 1AA	N/A	GIBRALTAR
1016589	Remote Bookmaker's Licence	TDCO LIMITED	TDCO LIMITED	. SIMON PATRICK HENRY LARKIN, . KEAN NEIL GRIVE	6TH FLOOR, 6 KEAN STREET	LONDON	WC2B 4AS	N/A	ENGLAND
1017375	Remote Bookmaker's Licence	NOVIGROUP LIMITED	NOVIBET	. EDWARD PEPPER, . LORNA RICHARDS, . PANAGIOTIS PITER TRATARIS	CLINCH'S HOUSE, LORD STREET	DOUGLAS IM99 1RZ	ISLE OF MAN	N/A	ISLE OF MAN
1017725	Remote Bookmaker's Licence	FITZWILLIAM SPORTS LIMITED	FITZWILLIAM SPORTS	MR JAY DILGER, MR PAUL BYRNE	38 FITZWILLIAM STREET UPPER	DUBLIN, D02 KV05, IRELAND	DUBLIN 2	DUBLIN CITY	NA
1017899	Remote Bookmaker's Licence	TONYBET OU	TONYBET	MISS JEKATERINA DANILOVA, MR DMITRY ARABULI	HARJU MAAKOND, LASNAMAE,	LINNAOSA, PUNANE TU 14A-4	KORRUS 41914, TALLINN 13619	N/A	ESTONIA

Licence Ref	Description	Licensee Name	Trading Name	Nominee Name	Address 1	Address 2	Address 3	Address 4	Address 5
1018051	Remote Bookmaker's Licence	TAICHI TECH LTD	FAFABET	. LIANG SONG, . JUN ZHU	SPACES	NO 9 GREYFRIARS ROAD	READING RG11NU	N/A	UK
1019015	Remote Bookmaker's Licence	TRADESPREADS EUROPE LIMITED	TRADESPREADS EUROPE LIMITED	MR KEITH BYRNE	77 LOWER CAMDEN STREET	NA	NA	DUBLIN CITY	NA
1019276	Remote Bookmaker's Licence	TERMINUS PLATFORM IRELAND LIMITED	1XBET	. SERGIO TORRES MOLINS, . YAGO MARCEL PERRIN VAZQUEZ	MESPIL BUSINESS CENTRE	MESPIL HOUSE	SUSSEX ROAD	DUBLIN CITY	NA
1020269	Remote Bookmaker's Licence	MALTIX LIMITED	SOFT2BET	. OLEKSII ZHYTNIK, . URI POLIAVICH	QUAD CENTRAL Q3 LEVEL 3	TRIQ L-ESPORTATURI, ZONE 1 CENTRAL BUSINESS DISTRICT	BIRKIRKARA CBD 1040	N/A	MALTA
1019252	Remote Bookmaker's Licence	GS BETTING LIMITED	GROUPSTAKES	MR KEVIN O'CALLAGHAN	GROUND FLOOR	71 LOWER BAGGOT STREET	NA	DUBLIN CITY	NA
1020267	Remote Betting Intermediary's Licence	BETDEX LABS INC	BETDEX LABS INC	. VARUN SUDHAKAR, . NIGEL JOHN ECCLES	2093 PHILADELPHIA PIKE	1476 CLAYMONT DELAWARE	19703	N/A	UNITED STATES

Licence Ref	Description	Licensee Name	Trading Name	Nominee Name	Address 1	Address 2	Address 3	Address 4	Address 5
1018081	Remote Bookmaker's Licence	BM SOLUTIONS LTD	BETMASTER	. DENIS SHADLOV, . JEAN NOEL AZZOPARDI	THE EDGE COURT	OFFICE 2, GUZE DUCA STREET	HAL QORMI QRM 9088	N/A	MALTA
1019666	Remote Bookmaker's Licence	MIDNITE EUROPE LIMITED	MIDNITE	. DANIEL AO QU, . NICHOLAS PEARSON WRIGHT	LEVEL 3 (SUITE 2327) TOWER BUSINESS	TOWER STREET	SWATAR BIRKIRKARA BKR4013	N/A	MALTA
1020488	Remote Bookmaker's Licence	MAKERS BOOK LIMITED	MAKERS BOOK	MR MICHAEL JOSEPH ELIAS, MR TURLOUGH MULLEN	CARTONS GARDEN	TOMSALLAGH	FERNS	CO. WEXFORD	NA
1020816	Remote Bookmaker's Licence	VELVIEW LTD	THE CRUCIBLE SNOOKER CLUB	MR FINBARR CORKERY	NICHOLAS WELL LANE	OFF BLARNEY STREET	CORK	CO. CORK	NA
1011367	Remote Bookmaker's Licence	CHIEFTAIN BOOKMAKERS LTD	CHIEFTAIN BOOKMAKERS LTD	MR PAUL DOYLE, MR JOHN SINNOTT JNR	UPPER MAIN STREET	FERNS	NA	CO. WEXFORD	NA
1011001	Remote Bookmaker's Licence	STAR SPREADS LIMITED	STAR SPREADS	MR BENJAMIN KEITH, RUSSELL CANDLER	UNIT 10E ENTERPRISE CENTRE	GOREY BUSINESS PARK, RAMSTOWN	GOREY	CO. WEXFORD	NA
1010146	Remote Bookmaker's Licence	PETFRE (GIBRALTAR) LIMITED	BETFRED	. RUSSELL YOUNG	5/2 WATERPORT PLACE	2 EUROPORT AVENUE	GIBRALTAR GX11 1AA	N/A	GIBRALTAR

Licence Ref	Description	Licensee Name	Trading Name	Nominee Name	Address 1	Address 2	Address 3	Address 4	Address 5
1020369	Remote Bookmaker's Licence	SLIGO LIMITED	SLIGO LIMITED	. ANDREW CASSAR, . YAKOV LERNER	64 EXCALIBUR	B BONTADINI	B'KARA BKR 1737	N/A	MALTA
1020364	Remote Bookmaker's Licence	JUNGLE X IE LIMITED	JUNGLE X IE LIMITED	. TAKEAKI KAWAKITA, . FUMITADA NAOE	WEWORK AVIATION HOUSE	125 KINGSWY	LONDON WC2B6NH	N/A	ENGLAND
1020437	Remote Bookmaker's Licence	COPYBET EU LTD	COPYBET	. VITALY AVTAYKIN, . ANTON IVANOV	KRINOU 3 OFFICE 701	THE OVAL	LIMASSOL, 4103	N/A	CYPRUS
1020669	Remote Bookmaker's Licence	SWIFTY SPORTS LIMITED	SWIFTY SPORTS LIMITED	DURAN TREVOR KRUMMECK	WORKHUB	51 BRACKEN ROAD	SANDYFORD BUSINESS PARK	DUBLIN CITY	NA
1012916	Remote Bookmaker's Licence	LOTTOMATRIX OPERATIONS LIMITED	JACKPOT.COM	. HADAR SIVAN . YARIV RON	LEVEL 3	10 LAPSI STREET	ST JULIANS STJ1261	N/A	MALTA
